# Exosome Source Matters: A Comprehensive Review from the Perspective of Diverse Cellular Origins

**DOI:** 10.3390/pharmaceutics17020147

**Published:** 2025-01-22

**Authors:** Yiru Chen, Weikun Qi, Zhenghao Wang, Feng Niu

**Affiliations:** Plastic Surgery Hospital, Chinese Academy of Medical Sciences and Peking Union Medical College, No. 33 Badachu Road, Shijingshan, Beijing 100144, China; chenyirudoctor@student.pumc.edu.cn (Y.C.);

**Keywords:** exosome, regenerative medicine, cellular origin, extracellular vesicle

## Abstract

Exosomes have emerged as promising therapeutic agents in regenerative medicine. This review introduces a novel cell type-oriented perspective to systematically analyze exosomal properties in regenerative therapies. To our knowledge, this review is the first to comprehensively compare exosomes based on cellular source type, offering unprecedented insights into selecting optimal exosome producers for targeted regenerative applications. Factors beyond cellular origin influencing exosomal therapeutic efficacy, such as donor sites and collection methods, are also explored here. By synthesizing key advances, we propose promising research directions in the end. We aim to accelerate the development of more effective exosome-based regenerative therapies and highlight underexplored directions in this rapidly evolving field.

## 1. Introduction

Exosomes, a subset of extracellular vesicles with a general size range smaller than 200 nm in diameter, secreted by almost all cell types [1,2], serve as intercellular communicators, and play critical roles in multiple physiological and pathological processes [3]. Specifically, exosomes can promote tissue repair and regeneration, which is mainly attributed to their ability to carry a variety of bioactive molecules and deliver them to target cells, low immunogenicity, and high biosafety [4,5]. These characteristics enable exosomes to demonstrate significant application potential in tissue engineering, wound healing, neurodegenerative disease treatment, and cardiovascular disease intervention [6,7].

The isolation and characterization of exosomes are crucial steps in exosomal research. To improve the reproducibility and quality of research in this field, MISEV (Minimal Information for Studies of Extracellular Vesicles) guidelines play a critical role in ensuring that scientists adopt standardized isolation methods [2].

Before clinical application, it is essential to select the cellular sources of exosomes based on the specific intention, while optimizing the potential influencing factors to maximize therapeutic effects. Growing numbers of exosome-related research are emerging, but there is no review to systematically compare exosomes from various cell types when considering applications in regenerative medicine. This study will provide a comprehensive and novel perspective by reviewing relevant studies in Pubmed, Web of Science, and Embase databases, focusing on a cell type-oriented approach. We aim to provide a reference for choosing appropriate cellular sources of exosomes for specific usage, bridging the gap between regenerative medicine and nanomedicine.

## 2. Production, Isolation, Modification, and Storage of Exosomes

### 2.1. Production

There are two primary sources of exosome production: biological fluid and cell culture medium. Extensively studied biological fluids include serum, platelet-rich plasma, urine, cerebrospinal fluid, breast milk, and saliva. The production yield varies depending on parent cell types and the conditions under which the cells are cultured, or the biological fluid is preconditioned. For example, MSCs produce substantial exosomes [8], while dendritic cells produce a more limited number [9]. Preconditioning conditions, including oxygen levels and growth factors, significantly influence production [10]. Stimulating factors related to the yield can be divided into biochemical, mechanical, and exosome structural engineering [11]. Optimizing these factors is crucial for high yields of production.

### 2.2. Isolation and Purification

Exosomes’ overlap in size, density, content, and biological markers has made obtaining high exosomal purity challenging. Most existing technologies can not completely distinguish exosomes from lipoproteins with similar biophysical or biochemical properties and extracellular vesicles from non-endosomal pathways, leading to a low purity of desired exosomes [12].

Commonly used techniques include ultracentrifugation, size-exclusion chromatography (SEC), polymer precipitation, immunoaffinity capture, and microfluidics-derived techniques [13]. Ultracentrifugation is widely used due to its high recovery rate, but the co-isolation of other vesicles and the time-consuming process limit its applications [12]. SEC utilizes the size difference to isolate exosomes. Their procedures could largely preserve the integrity and bioactivity of exosomes but could not separate exosomes from other similar-size vesicles [14]. Every single technique above has advantages and disadvantages. Therefore, scientists have been trying to combine multiple complementary methods and found that the combination performs better in reducing contamination, improving separation purity, and maintaining the natural characteristics of exosomes [15].

### 2.3. Modification

**Figure 1:**
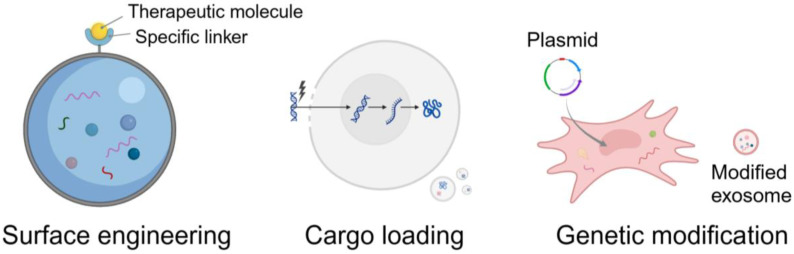
Three main strategies to modify exosomes (created with BioRender.com).

Three main strategies for modifying exosomes are surface engineering, cargo loading, and genetic modification (Figure 1), with the goal of enhancing the therapeutic potential, targeting specificity, and stability.

Surface engineering involves modifying the exosomal membrane to improve targeting capabilities, circulation time, and uptake by specific cell types. Many scientists have summarized various surface engineering strategies, which can be categorized into modification at the cellular level and modification after exosomal isolation, or chemical and biological strategies [16,17,18]. For instance, Yu F et al. have engineered adipose stem cell-derived exosomes (ADSC-Exos) with specific peptide linkers to conjugate therapeutic molecules and have obtained ideal anti-inflammatory and tissue regenerative effects [19].

Cargo loading refers to incorporating therapeutic effector molecules, such as drugs, RNA, or proteins, into exosomes for specific purposes. These methods have also been comprehensively summarized by various studies [20,21]. It can be categorized into active loading and passive loading. Active loading normally means employing techniques such as electroporation, sonication, or extrusion to enhance cargo incorporation. Passive loading often refers to incubating exosomes with the desired cargo, allowing for diffusion-based incorporation, which includes co-culture, transfection, and incubation.

Genetic modification entails manipulating donor cells to express particular proteins or RNAs that are subsequently incorporated into exosomes. It can be divided into modification of parent cells and surface display via genetic engineering [22]. By transfecting donor cells with genes encoding desired therapeutic agents, these cells can produce exosomes enriched with specific proteins or RNAs. For instance, engineering cells to express therapeutic mRNA or siRNA enables the secretion of exosomes loaded with these genetic materials, facilitating targeted gene therapy applications [23].

### 2.4. Storage

To ensure the repeatability of studies on exosomal structure, content, and function, while keeping exosomes easy to transport and handle, scientists have developed three main storage techniques: cryopreservation, freeze-drying, and spray-drying.

For cryopreservation, exosomes are commonly stored at a temperature of 4 °C or −196 °C [12], but they might be negatively affected by repeated freezing and thawing [24]. Freeze-drying removes water from the exosome samples under low pressure [25], which stabilizes the exosomes and makes them easier to reconstitute. When lyophilized, exosomes can be stored at room temperature for extended periods. However, the type of cryoprotectants is crucial to prevent aggregation and ensure the exosomes’ functional integrity upon reconstitution [26]. Spray-drying converts the liquid exosome suspension into a dry powder by rapidly drying it with hot gas, offering easy transportation [12]. But high temperatures potentially denature sensitive proteins on the exosome surface, making it less suitable for exosomes with heat-sensitive components [27].

## 3. Factors Influencing the Efficacy of Exosome-Based Applications in Tissue Regeneration

**Figure 2:**
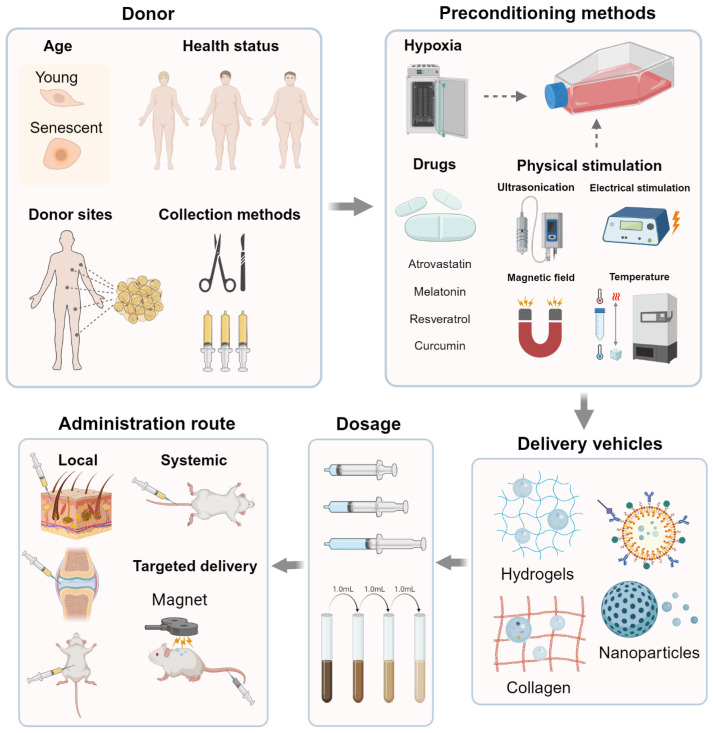
Different factors that may influence the exosome-based therapeutic effects (created with BioRender.com).

Several other factors critically affect the therapeutic outcomes of exosome-based interventions, including donor cell condition, dosage, administration route, and delivery vehicles used (Figure 2).

### 3.1. Donor Cell Condition

Age, health status, donor sites, collection methods, and preconditioning methods of donor cells are non-negligible factors that affect the therapeutic outcomes.

Aging in donor cells generally leads to a decline in the quality of exosomes, which usually manifests as reduced anti-inflammatory and regenerative capabilities. For instance, exosomes derived from older BMSCs exhibit diminished effects in osteogenic and lipogenic abilities compared to those derived from younger BMSCs [28]. In addition, cells with different health statuses may produce exosomes with significant differences in morphology, function, or molecules. Cells derived from diseased individuals produce exosomes with altered molecular profiles, leading to impaired functionality or adverse effects. Oncoproteins, including EGFR, GRB2, and SRC, have been reported to be enriched in the non-small cell lung cancer (NSCLC) exosomes, which promote the cancer’s development [29]. Different sites and collection methods may also lead to exosomes with varying traits. Visconte C et al. reported that abdominal adipose tissue obtained by surgical excision and lipoaspiration might produce exosomes with distinct characteristics [30]. They also demonstrated that exosomes released by ADSCs from different anatomical locations contained a different abundance of miRNAs. Preconditioning strategies, such as hypoxia or cytokine stimulation, can change the regenerative potential of donor cells and their exosomes. Hypoxia could upregulate pro-angiogenic factors in MSCs, improving exosomal abilities to promote neovascularization and tissue repair in mouse models of femoral fracture [31]. Preconditioning methods with rapamycin and growth factor deprivation have also been proven to increase the release of exosomes [32].

### 3.2. Dosage

Higher exosomal doses generally lead to a more pronounced regenerative effect; however, there is a threshold beyond which additional exosomes may not provide further benefits or may even cause adverse effects (Table 1). In traumatic brain injury (TBI) models, researchers found that compared with 50 μg or 200 μg groups, 100 μg exosomes per rat showed more significant efficacy in improving sensory-motor and cognitive function, reducing hippocampal neuron cell loss and promoting neurogenesis [33]. The therapeutic dose of exosomes commonly ranges from 10 to 100 μg of protein in mouse models [34], but evaluation of the effective dose in specific conditions is lacking.

### 3.3. Administration Route

The route of exosomal administration substantially affects exosomal capabilities [41]. The most frequently applied routes include systemic (intravenous), local (e.g., intra-muscular, intra-articular, or intra-dermal), and targeted delivery methods.

Systemic administration is usually applied to deliver exosomes throughout the body, targeting multiple sites simultaneously. Although it is beneficial in treating conditions where widespread distribution is needed, rapid clearance by the liver, spleen, and kidneys decreases the therapeutic efficacy, and non-specific uptake by off-target tissues can reduce the concentration of exosomes at the desired site [42]. In contrast, the biggest advantage of local administration is the high exosomal levels at the target site and the low risk of systemic side effects [43]. This route is commonly used for conditions where localized action is desired [44]. However, repeated administration procedures might be required to maintain adequate levels at the site. Targeted delivery methods, such as ligand-modified exosomes or magnetic targeting, enable precise delivery to diseased tissues [45]. Maximizing efficacy while minimizing off-target effects, they have become attractive research hotspots. Superparamagnetic iron oxide nanoparticles (SPIONs) possess excellent magnetic properties and can be concentrated using an external magnetic field [46]. Combining the magnetic targeting properties of SPIONs with the homing abilities of exosomes, scientists have achieved a “dual targeting” effect and excellent therapeutic efficacy in NSCLC treatment [47].

### 3.4. Delivery Vehicles

To enhance the effectiveness and address the drawbacks of existing therapies, growing numbers of studies have been exploring new materials as delivery vehicles.

Hydrogels are effective delivery systems due to their sustained release, biocompatibility, and biodegradability [48]. They can be engineered to respond to environmental stimuli, such as pH or temperature, to achieve controlled exosome release [49]. Nevertheless, controlling the hydrogels’ degradation rate to align with the tissue regeneration process is a crucial challenge. Functionalized with ligands, nanoparticles (NPs) become practical tools to protect exosomes from rapid clearance [50]. Nonetheless, targeted ligands may trigger an immune response, and certain NP materials’ biocompatibility and potential toxicity remain significant hurdles [51]. Various scaffold materials are used as exosomal delivery platforms to provide controlled release. Collagen-based scaffolds are frequently chosen due to collagen’s role as a major component of the extracellular matrix (ECM) and its natural involvement in tissue repair processes. Promoting cell adhesion and proliferation notwithstanding, their mechanical properties may be inadequate in load-bearing tissues [52]. In these scenarios, the lack of long-term structural integrity in collagen can hinder the sustained bioactive function of exosomes at the injury site.

## 4. Exosomes from Different Cellular Sources

**Figure 3:**
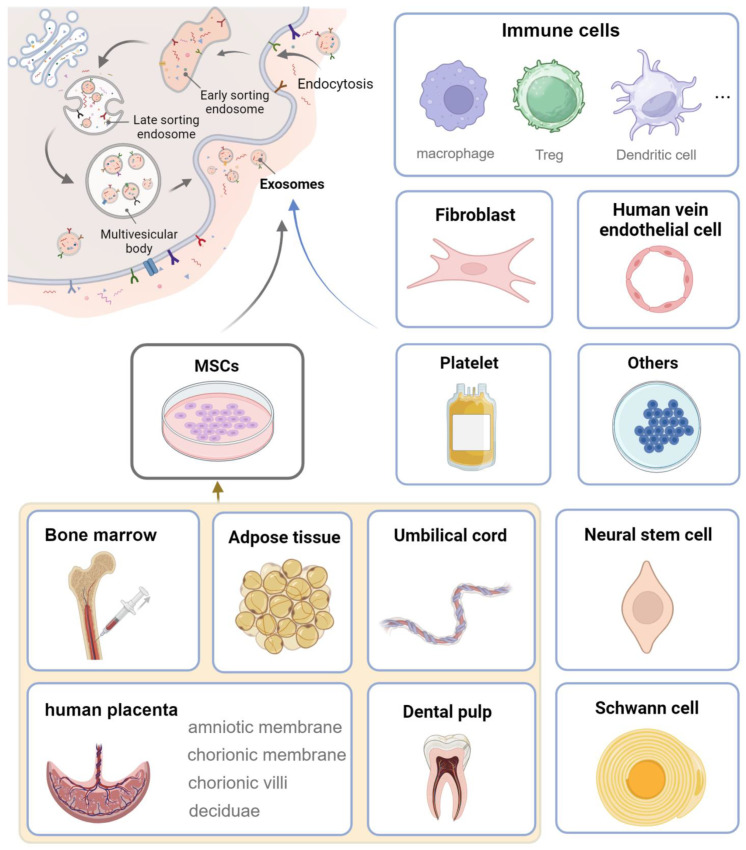
Cellular sources of exosomes that are widely researched in regenerative medicine (created with BioRender.com).

The regenerative potential of exosomes is intricately tied to their cellular origin, with each source imparting unique properties and functionalities to the secreted vesicles. This cellular specificity not only underlies the diverse applications but also highlights the need for a thorough understanding of how different sources of exosomes contribute to tissue restoration. Currently, several cellular sources have been broadly studied (Figure 3), such as MSCs, immune cells, neural stem cells (NSCs), fibroblasts, umbilical vein endothelial cells (UVECs), and Schwann cells (SCs).

### 4.1. MSC-Derived Exosome

MSCs are a diverse group of multipotent stromal cells capable of differentiating into various cell types, which is also evident in their exosomes (MSC-Exos) [53].

Extensively studied MSC types as sources of exosomes include bone marrow MSC (BMSC), ADSC, umbilical cord MSC (UCMSC), placental MSC (PMSC), and DPSC.

#### 4.1.1. BMSC-Derived Exosome

BMSC-derived exosomes (BMSC-Exos) have attracted research attention since the early years, correlated studies are relatively more extensive than other types of exosomes [54].

For wound healing, BMSC-Exos outperform exosomes derived from ADSC and UCMSC in enhancing the proliferation of dermal fibroblast (DF) [55]. Pomatto M et al. reported that, unlike ADSC-derived extracellular vesicle proteins, proteins uniquely enriched in BMSC-derived extracellular vesicles (BMSC-EVs) are associated with cell adhesion, glycolysis, and fructose–galactose metabolism, suggesting that BMSC-Exos mainly promote cell proliferation [56]. However, their results indicated that BMSC-Exos might be inferior to ADSC-derived exosomes (ADSC-Exos) in achieving optimal effects, possibly due to insufficient angiogenic abilities. This conclusion contrasts with the later findings of Soni et al., who demonstrated that BMSC-Exos have superior angiogenesis-promoting capabilities [57].

BMSC-Exos exhibit a high propensity to promote bone tissue regeneration due to their enrichment in osteogenic factors like microRNA-148a and long non-coding RNA-H19 [58,59]. Additionally, studies have shown that BMSC-Exos possess enhanced immunomodulatory effects, creating a favorable microenvironment for healing [57]. In addition, their beneficial effects in other fields such as cardiovascular diseases and neural regeneration were also reported [60,61].

However, obtaining BMSCs from primary sources involves an invasive and painful procedure, which poses a notable disadvantage when contrasted with more readily accessible sources of MSCs [62,63]. This invasiveness can limit the scalability of BMSC-Exo production and may reduce donor willingness. BMSC-Exos are also highly susceptible to age-related changes in their parent cells. As donor age increases, their regenerative capacity and therapeutic efficacy may decline more significantly than exosomes from other MSC sources [64].

Apart from the primary sources mentioned above, it is essential to develop sustainable and scalable alternatives such as cell lines. Commonly used BMSC lines are immortalized cell lines like hTERT-BMSC (human telomerase reverse transcriptase-BMSC) [65], which exhibit extended proliferative capacity. Though more scalable, immortalized BMSC lines may differ phenotypically and functionally from their primary counterparts, raising concerns about their translational relevance [66].

#### 4.1.2. ADSC-Derived Exosome

ADSC exhibits shorter population doubling time and stronger antiapoptotic potential than other types of MSC [67]. Studies have also indicated that ADSCs yield more exosomes with simpler isolation techniques and lower invasiveness [68]. Furthermore, the increasing prevalence of overweight and obesity due to improved modern living standards has made acquiring adipose stem cells more feasible.

A distinctive feature of ADSC is higher lipid content. The elevated lipid composition contributes to their enhanced paracrine signaling capacity, mediated by exosomes. They promote tissue repair through increased cellular communication, improved membrane fusion, and better interaction with target cells [69]. Enriched with anti-inflammatory cytokines, growth factors, and miRNAs, ADSC-Exos can modulate the local immune environment and promote angiogenesis and adipogenesis [70]. Their anti-fibrotic effects were also observed, which is beneficial for scar reduction [71].

Emerging evidence suggests that the source of adipose tissue used to derive ADSCs significantly impacts the properties of the resulting exosomes. Studies examining ADSCs from various fat depots, such as subcutaneous, visceral, perivascular, and infrapatellar fat, have revealed depot-specific variations in exosomal composition and therapeutic efficacy. For instance, the expression levels of key chondrogenic and osteogenic genes in cells isolated from infrapatellar fat pad (IPFP) were proved to be higher than those from subcutaneous adipose tissue, indicating IPFP might be a better source for ADSC-Exos for cartilage and bone regeneration [72]. For subcutaneous adipose tissue, there are also variations between different anatomical locations [73]. These depot-specific differences further highlight the versatility for customization of ADSC-Exos. Despite the abundance of sources, ADSC-Exos from elder individuals may be less effective than those from younger individuals in terms of therapeutic outcomes, as ADSCs derived from different age ranges might have significant differences in function [74].

In addition, ADSC-Exos were also revealed to possess therapeutic effects on inflammation-related diseases (e.g., Crohn’s disease, arthritis, etc.), myocardial ischemia, hair loss, and delayed photoaging [75,76,77].

Primary cultures of ADSCs were indicated to be inferior to their commercially obtained counterparts in cell growth [78]. Regenerative capabilities of immortalized adipose-derived mesenchymal stem cell lines have been revealed [79], but their application in exosomal research needs more investigation.

#### 4.1.3. Human UCMSC-Derived Exosome

In recent years, there has been a surge in research exploring the therapeutic potential of human UCMSC-derived exosomes (hUCMSC-Exos).

Firstly, the collection methods of hUCMSCs are much simpler than those for most other MSC sources. Derived from umbilical cord blood, hUCMSCs can be obtained through a painless procedure simply by extracting blood from the umbilical cord. In contrast, harvesting MSCs from alternative sources such as bone marrow or adipose tissue typically involves invasive techniques.

Existing studies have exhibited superior regenerative properties of hUCMSC-Exos. A study analyzing exosomes derived from BMSCs, ADSCs, and hUCMSCs demonstrated the greatest capacity of hUCMSC-Exos to promote keratinocyte migration [55]. Scientists have conducted a systematic analysis of protein components in these three types of exosomes, indicating that hUCMSC-Exos are more prominent in tissue repair [80].

Due to the notable immunomodulation ability, hUCMSC-Exo is regarded as an effective strategy for treating inflammatory or autoimmune diseases. Applications of hUCMSC-Exos in inflammatory bowel disease have been a research hotspot in recent years [81,82]. Periocular injections of hUCMSC-Exos have shown substantial inhibition of autoimmune uveitis progression in rats [83]. Moreover, complete subacute spinal cord injury (SCI) patients after intrathecal injection of hUCMSC-Exos have observed functional improvements [84]. These exosomes can also obviously mitigate graft-versus-host disease injury by alleviating the oxidative–reductive metabolic dysfunction [85].

However, several issues must be considered. Maternal factors such as maternal obesity and diabetes have been widely studied. Maternal obesity influences the metabolism and bioenergetic profile of hUSMSCs [86], while gestational diabetes mellitus (DM) might contribute to premature aging and mitochondrial dysfunction [87]. Additionally, immortalized hUCMSCs have been reported to exert therapeutic influence in other fields [88], but the use of them in exosomal research remains blank.

#### 4.1.4. Human PMSC-Derived Exosome

The human placenta serves as a crucial source of MSC-Exos. The availability of this tissue is well established, as the placenta is typically discarded as medical waste after birth and can be readily obtained with minimal ethical concerns [89]. PMSCs have attracted extensive attention due to their unique biological characteristics. However, there is still no widely recognized “standard” commercial immortalized PMSC cell line. Therefore, most of the existing studies on PMSCs use cells that are directly isolated from placental tissue.

The placenta tissue can be divided into four layers: amniotic membrane (AM), chorionic membrane (CM), chorionic villi (CV), and deciduae (DC) [90]. Each layer yields distinct MSCs: AMMSCs, CMMSCs, CVMSCs, and DCMSCs, respectively. Current research focusing on human AMMSC-derived exosomes (AMMSC-Exos) is relatively abundant, whereas studies on others remain limited.

##### Human AMMSC-Derived Exosome

AMMSCs and their secretome have consistently exhibited immune-regulatory capabilities, such as suppressing T cell proliferation induced by alloantigens and shifting the macrophage 1 (M1)/macrophage 2 (M2) ratio of synovial macrophages in osteoarthritis [91]. AMMSC-Exos can also reduce the number of Kupffer cells and the levels of inflammatory cytokines in nonalcoholic steatohepatitis rat models [92]. Although a few studies have directly examined AMMSC-Exos alongside exosomes from other cell types, comparisons between their parent cells offer valuable insights into the potential of AMMSC-Exos. Topoluk N et al. demonstrated that AMMSCs possess stronger chondroprotective abilities than ADSCs and can effectively mitigate cartilage damage induced by macrophages, an effect not observed with human ADSCs [93].

A significant advantage of AMMSC-Exos is their small particle size, with a mean diameter of 72 nm, notably smaller than the mean sizes of ADSC-Exos (220 nm) and UCMSC-Exos (120 nm), facilitating their passage through physiological barriers [94].

For nervous system regeneration, AMMSC-Exos were found to effectively enhance neuron survival by regulating cell apoptosis and improving the neurobehavioral function of cerebellar palsy rat models [95]. In acute traumatic spinal cord injury (TSCI) rats, human AMMSC-Exos were shown to significantly reduce the lesion volume [96].

In wound healing, scientists have found that combining acellular amniotic membrane scaffolds with ADSC-Exos significantly enhances the therapeutic effects on diabetic wound healing compared to the single application of ADSC-Exos [97]. Subsequently, Noh CH et al. demonstrated that exosome-rich conditioned medium from AMMSCs improved whole-skin-excision rats’ wound healing, with rapid wound closure and reduced scar tissue formation [94].

##### Other Human PMSC-Derived Exosome

Research correlated with exosomes from CMMSCs (CMMSC-Exos), CVMSCs (CVMSC-Exos), or DCMSCs (DCMSC-Exos) is limited, but the existing studies could provide a reference for future exploration.

CMMSC-Exos could be absorbed by multiple cells (including MSCs derived from synovial fibroblasts, osteoblasts, and periosteum) isolated from tissues associated with osteoarthritis, suggesting their potential in the treatment of osteoarthritis [98]. CVMSC-Exos have shown anti-cancer effects on ovarian cancer cells and promote trophoblast migration and proliferation [99]. Salomon C et al. isolated CVMSC from placental villi and further obtained CVMSC-Exos from acellular CVMSC, finding that CVMSC-Exos contribute to placental adaptation to low oxygen [100]. Zhang CP’s group provided evidence for the regenerative effects of DCMSC-Exos, they revealed that DCMSC-Exos can promote the proliferation, migration, and differentiation of high-glucose-induced senescent fibroblasts [101].

#### 4.1.5. DPSC-Derived Exosome

DPSCs can be obtained during routine dental procedures [102] and exhibit robustness under cryopreservation [103]. Exosomes derived from DPSCs (DPSC-Exos) possess higher drug-loading efficiency than many other MSC-derived exosomes [104]. Notably, unlike the common effect of aging on the regenerative ability of exosomes, senescent DPSC-derived exosomes can improve the antioxidant ability, proliferation, migration, and survival rate of young DPSCs [105].

DPSC-Exos demonstrate exceptional efficacy in oral diseases, surpassing other exosomes. When isolated from odontogenic differentiation culture conditions, DPSC-Exos can increase the expression of genes necessary for odontogenic differentiation in vitro and promote dental pulp regeneration in vivo [106,107]. They were also proven to have stronger immunoregulation activity, anti-necrosis, and anti-apoptosis capabilities than BMSC-Exos [108,109]. Shen Z et al. claimed that combining DPSC-Exos with chitosan hydrogel effectively inhibited periodontal inflammation and reduced epithelial damage and alveolar bone loss [110], consistent with Zheng J et al. [111].

In nervous system regeneration, DPSC-Exos were found to reduce neuronal apoptosis by transferring miR-877–3p, improving brain edema and infarct volume in cerebral ischemia–reperfusion injury (I/R) rat models [112]. Liang X et al. reported that DPSC-Exos attenuated the neuroinflammation and microglial pyroptosis in subarachnoid hemorrhage through miR-197-3p/FOXO3 axis [113]. In addition, they can enhance sciatic nerve regeneration by increasing the secretion of neurotrophic factors, proliferation, and migration of SCs [114].

DPSC-Exo is also widely studied in other areas such as bone-related realms, flap transplantation, and wound healing [115,116]. Compared with BMSC-Exos and ADSC-Exos, isolating sufficient quantities of DPSC-Exos remains a significant challenge, which needs more innovative technologies [117].

Similarly, commercialized or immortalized DPSC lines have been researched in some fields [118,119]. But the tumorigenic potential and long-term influence are not fully explored and there is a long way before applying these cell lines to an exosome-related field.

### 4.2. Immune Cell-Derived Exosome

The overall body environment is nonnegligible for tissue regeneration. Immune modulation is a key entry point for treating inflammation and autoimmune diseases. While MSC-Exos exhibit certain immunomodulatory properties, immune cells serve as a potential source of exosomes with more specific effects [120].

In contrast to MSC-Exos, the potential effects of immune cell-derived exosomes still harbor many unknowns. There has been relatively more literature on exosomes derived from macrophages and T cells.

#### 4.2.1. Macrophage-Derived Exosome

Macrophages exist in various activation states (e.g., M1 pro-inflammatory and M2 anti-inflammatory), with different contents and functions of exosomes. M1 macrophages and their exosomes are known for pro-inflammatory effects, which might adversely affect tissue regeneration [121]. Therefore, related research on M1 macrophage-derived exosomes is limited. Meanwhile, M2 macrophages are widely acknowledged to enhance inflammation regression and tissue repair. Exosomes derived from them (M2-Exos) replicate multiple functions of M2 macrophages [122], exhibiting promising immunomodulation properties. In this section, we will review the applications of M2-Exos in regenerative medicine.

M2-Exos possess a strong capability to guide the phenotypic switch of M1 to M2 macrophages, providing a promising strategy for the therapy of diseases associated with imbalances of pro-inflammatory and anti-inflammatory responses. Due to this, they were revealed to facilitate diabetic fracture healing [122]. CCL24 and MFG-E8, abundant in M2-Exos, are key regulators of this switch, promoting wound repair by strengthening angiogenesis and epithelialization [123]. In addition, M2-Exos can enhance tendon-to-bone healing in aged rats by alleviating cellular senescence and improving the chondrogenic potential of BMSCs [124]. Other properties, such as promoting functional recovery after SCI, have also been reported [125].

In addition to primary macrophage cultures mentioned above, macrophage cell lines, such as the commonly used THP-1, have also been employed to generate exosomes for various research [126]. They are robust and easy to culture, offering a more consistent and reproducible source of exosomes compared to primary macrophages, which are often subject to donor variability and difficult to maintain over extended periods. For instance, RAW 264.7-derived M2-Exos have been reported to facilitate osteogenesis and promote bone regeneration after specific modification [127,128].

Notably, some miRNAs contained in M2-Exos may have unintended effects on tumor biology. For example, some M2-Exos content can increase tumor metastasis and progression [126,129], requiring a more comprehensive investigation.

#### 4.2.2. T Cell-Derived Exosome

T cells, a critical component of the immune system, encompass various subtypes. Regulatory T cell (Treg)-derived exosomes (Treg-Exos) possess potent immunomodulatory properties and have a strong research foundation in regenerative medicine [130].

Treg-Exos exhibit remarkable potential in modulating immune responses during tissue repair processes. Their ability to suppress effector T cells and promote tolerance makes them particularly valuable in scenarios where fine-tuning the immune system is crucial for optimal healing [120].

The application of Treg-Exos in transplantation rejection has been researched widely. In the humanized mouse skin transplantation model, human Treg-Exos were observed to inhibit homologous immunity-mediated skin tissue damage by reducing immune cell infiltration and prolonging skin allograft survival [131]. Beyond this, Treg-Exos can also prolong the survival time of rat kidney transplantation models [132] and rat orthotopic liver transplantation models [133].

Moreover, Treg-Exos were found to exert beneficial effects in the field of neural injuries, wound healing, and cardiovascular diseases. Yang C et al. demonstrated that Treg-Exos could prevent the apoptosis and inflammation of BV-2 microglia induced by oxygen–glucose deprivation/reperfusion [134]. Treg-Exos could also promote the phenotypic switch of macrophages, facilitating angiogenesis and tissue remodeling for successful diabetic wound healing [135]. For acute myocardial infarction, Treg-Exos were observed to improve cardiac function by promoting macrophage M2 polarization [136].

Several challenges must be addressed. For example, Tregs can be categorized into two types with distinct properties: those directly derived from the thymus and peripheral-derived Tregs (pTregs) [134]. Similar variations in Treg cell source types and isolation methods of Tregs and exosomes may lead to heterogeneity of the effects of Treg-Exos. Additionally, stable Treg cell lines that can be used for exosomal research are still lacking, and relevant studies are limited.

### 4.3. NSC-Derived Exosome

NSC-Exos have also become a promising focus. One of the most famous properties is their neuroregenerative effect. Apparent decreases in infarct size and brain atrophy in murine thromboembolic stroke models were observed after applying NSC-Exos [137], effects not typically observed with MSC-Exos [138]. They can also mediate autophagy to inhibit neuroinflammation and promote functional recovery in SCI model rats at an early stage [139]. Furthermore, their function to promote neural recovery was reported to surpass those of MSC-Exos [137].

Another unique advantage is their potential to improve the integrity of the blood–brain barrier (BBB). Liu Y et al. established a BBB model using 5 × FAD primary cerebral endothelial cells and NSC-Exos, which reversed BBB defects caused by Alzheimer’s disease [140].

Beyond neuroregenerative effects, NSC-Exos were found to support wound repair and might play roles in controlling systemic aging speed. These exosomes contain abundant neurotrophic factors such as neuron-derived neurotrophic factor (NDNF) and immunoregulation proteins [141]. Zhang Y et al. transplanted healthy hypothalamic NSCs into the aging brain, finding that the exosomal miRNAs from these cells can decelerate aging [142].

A few research studies in other fields have used commercial or immortalized NSC lines, but primary NSCs remain the most widely studied source of exosomes currently. Nevertheless, establishing a stable amplification system without heterologous components and defining a method for single-exosome NSC analysis are still needed to improve NSC-Exos application [143].

### 4.4. Exosome Derived from Other Cellular Origins

#### 4.4.1. Fibroblast-Derived Exosome

Fibroblasts are indispensable for the body’s response to injuries, the synthesis and remodeling of the local ECM, and the formation of new tissue—processes in which exosomes produced by them (FC-Exos) play a crucial role [144]. For FC-Exos, exosomes can be obtained from primary fibroblasts and commercial or immortalized cell lines.

Compared with MSCs, primary fibroblasts can be isolated from the skin through less invasive procedures and are more closely associated with wound healing [145]. Scientists have identified several mechanisms underlying FC-Exos’ beneficial effects on wound healing, such as accelerating local collagen deposition and maturation [146]. In neural regeneration, primary fibroblasts derived from the sciatic nerve of rats were observed to enhance axonal regeneration and contribute to Schwann cell-mediated myelination [147,148,149].

Commercial or immortalized cell lines of fibroblasts have been extensively studied. They were found to possess antioxidant activity and could prevent ultraviolet B-induced senescence, indicating excellent potential as an anti-photoaging strategy [150]. Hu S et al. compared the anti-skin-aging capabilities of exosomes derived from three-dimensional spheroids of human DFs (3D HDF-XOs) with those of MSC-Exos, finding that 3D HDF-XOs are more effective in reducing skin aging [151]. Jang YN et al. demonstrated that human DF-neonatal-derived exosomes might exert anti-inflammation activity and improve the recovery of damaged skin barrier in atopic dermatitis [152].

#### 4.4.2. Human Umbilical Vein Endothelial Cell-Derived Exosome

Exosomes derived from human umbilical vein endothelial cells (HUVEC-Exos) have been applied in many regenerative areas. At present, HUVECs in the majority of existing studies were purchased from suppliers, which facilitates their exosomal research.

One of the most extensively studied applications is cutaneous repair. Scientists reported that HUVEC-Exos could facilitate diabetic wound healing when pretreated under hypoxic conditions or cooperated with bioengineered scaffolds [153]. Interestingly, when pretreated with advanced glycation end products to mimic conditions in DM, HUVEC-Exos were found to delay wound healing by modulating fibroblast autophagy [154]. In common skin wounds, apoptotic HUVEC-Exos could promote skin repair by increasing angiogenesis [155]. However, contrasting findings by Qi L et al. suggested that the exosomal miR-106b, which diminished the adhesion and viability of fibroblasts and keratinocytes, might harm wound healing [156].

For pro-angiogenic function, Guo L et al. demonstrated that pretreating HUVEC-Exos with an appropriate centration of H2O2 can enhance the angiogenic ability of endothelial progenitor cells, leading to increased skin flap survival [157]. Maiullari F et al. explored a 3D bioprinting strategy and revealed that HUVEC-Exos could be potentially employed as bioadditives for the formulation of bioinks, supporting the formation of a new functional vasculature when being loaded onto the bioprinted 3D structures [158]. Regarding neuroprotective effects, HUVEC-Exos have been proven to attenuate inflammation and apoptosis of neural cells [159] and protect nerve cells against I/R injuries [160]. In bone regeneration, HUVEC-Exos can drive osteogenic differentiation and boost the migratory potential of BMSCs [161].

There are still many unknowns. For example, some characteristics of HUVEC-Exos remain controversial as mentioned previously (e.g., the effects on wound healing). For preconditioning methods, contrary to the common belief that longer treatment times yield stronger effects, a 3-h hypoxic treatment had a similar or even weaker impact on HUVEC exosomes compared to a 15 min treatment [162].

#### 4.4.3. Schwann Cell-Derived Exosome

Due to the close relationship with the nervous system, exosomes derived from SCs (SC-Exos) have garnered significant attention in neural treatment.

In existing exosome-related studies, primary SCs could be isolated from sciatic nerves or derived from skin precursors (SKPs). For peripheral nerve regeneration, Lopez-Verrilli MA et al. first reported in 2013 that SC-Exos significantly increased axonal regeneration in vitro and enhanced the regenerative capacity of the post-injury sciatic nerve in vivo [163]. Subsequent research showed that this effect might be primarily produced by exosomes derived from repair Schwann cells (rSCs) rather than differentiated Schwann cells (dSCs) [164]. Moreover, exosomes derived from SKP-derived SCs could enhance the survival and repair of sensory neurons after oxygen–glucose deprivation (OGD) exposure [165], facilitate axon regeneration [166] and alleviate denervation-induced muscle atrophy [167].

Although SCs are an important component of PNS, their exosomes could also affect the regeneration after SCI. Pan D et al. identified that SC-Exos could induce axonal protection after SCI by enhancing autophagy and reducing apoptosis [168]. Consistent with this, Xu B et al.’s study showed that SC-Exos could activate mitophagy mediated by the AMPK pathway and improve mitochondrial dysfunction and necroptosis after SCI [169]. In bone tissue engineering, hydrogel-encapsulated SC-Exos optimized the microenvironment for bone regeneration by improving innervation, immunoregulation, angiogenesis, and osteogenesis [170].

Li Z et al. used rat cell lines of SCs (RSC96) and observed promoting dental pulp regeneration effects of their exosomes [171]. In addition, their multiple beneficial regenerative effects were also revealed in the in vitro model of cyclic mechanical strain (CMS)-induced dorsal root ganglion (DRG) cell injury [172].

It is undeniable that some properties of SC-Exos limit their application. For example, because of their terminal status, the culturing of primary SCs is complex and costly [173]. Moreover, SC-Exos’ regenerative capabilities might decrease after remaining denervated without axonal contact for a prolonged period, which needs more exploration and improvement [174].

## 5. Future Insights

Table 2 consolidates all the examples of exosome applications provided in the above text. There are many other cellular sources of exosomes showing regenerative effects, such as cardiosphere-derived cells (CDC) and hepatocytes. For instance, CDC-derived exosomes (CDC-Exos) could improve cardiac function after acute myocardial infarction [175]. Extracellular vesicles produced by hepatocytes were proven to promote liver regeneration [176].

**Figure 4:**
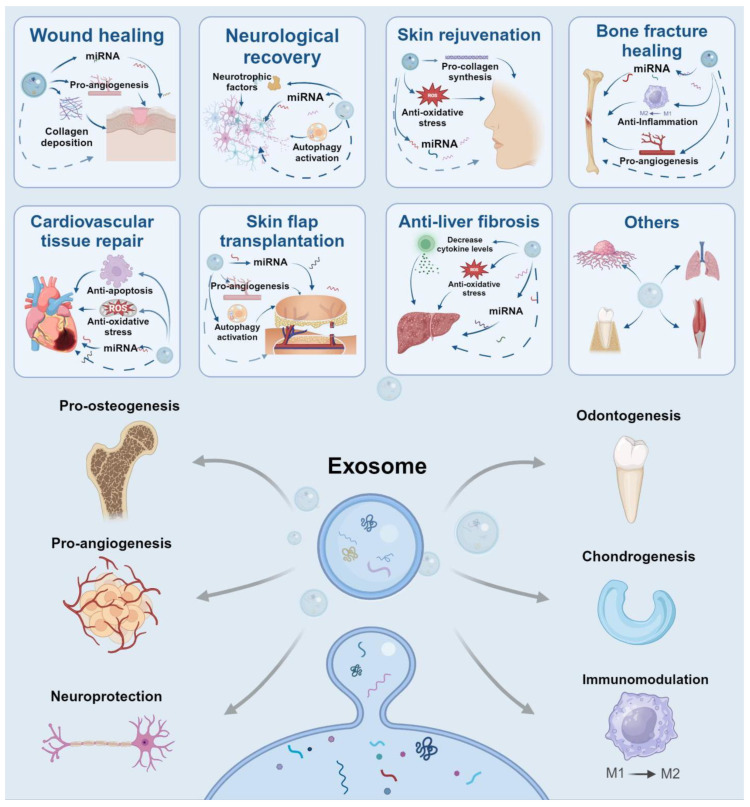
Characteristics of exosomes and their potential applications (created with BioRender.com) (miRNA: microRNA, ROS: reactive oxygen species, M1: macrophage 1, M2: macrophage 2).

In general, exosomes offer new possibilities for tissue regeneration across a wide range of medical conditions (Figure 4). Firstly, their acellular nature mitigates many safety concerns associated with cell transplantation [177]. Secondly, exosomes can cross biological barriers due to their nano-size, more efficiently than larger particles or cells to reach target tissues [178]. Thirdly, exosomes exhibit lower immunogenicity than whole cells, reducing the risk of immune rejection and potentially allowing for allogeneic applications [179]. These characteristics significantly broaden the donor pool and increase the scalability of exosome-based therapies. So far, researchers have compared the properties of different cellular exosomes in specific conditions. Table 3 shows studies comparing different types of exosomes, aiming to provide a reference for choosing the cellular origin of exosomes.

**Figure 5:**
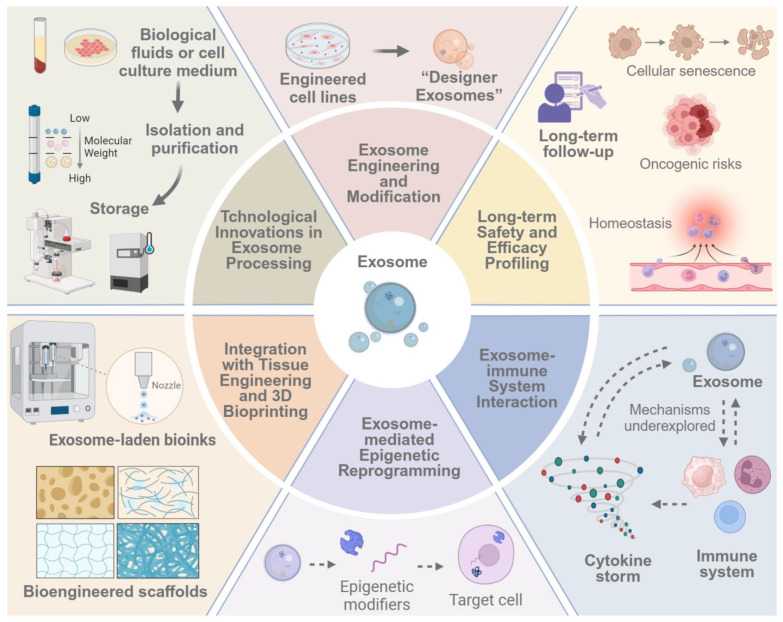
Underexplored exosomal directions in regenerative medicine (created with BioRender.com).

Several promising but underexplored strategies in exosome research warrant further investigation (Figure 5).

Firstly, technological innovations in exosome isolation, characterization, and large-scale production methodologies represent a critical area for advancement. Current techniques often yield heterogeneous exosome populations, potentially confounding research findings and limiting therapeutic efficacy. Developing novel approaches for high-purity and high-yield exosome isolation is crucial for research and clinical applications. Additionally, establishing standardized protocols for exosome characterization, encompassing physical and molecular attributes, is essential for ensuring reproducibility across studies and translating research findings into clinical practice.

While promising, current exosome modification methodologies are often limited in their precision and scalability. Future investigations should focus on developing techniques for fine-tuning exosome surface proteins and internal cargo with unprecedented specificity. This could involve the integration of synthetic biology principles, such as using engineered cell lines or artificial exosome-mimetic nanovesicles, to produce exosomes with tailored therapeutic properties. Such advancements may lead to the creation of “designer exosomes” capable of addressing specific regenerative challenges with enhanced efficacy and reduced off-target effects.

While the immunomodulatory properties of certain exosome populations have been documented, our understanding of the complex interplay between exosomes and various immune cell subsets remains limited. Delineating the mechanisms by which exosomes modulate immune responses in different tissue microenvironments could inform the development of exosome-based therapies that promote tissue regeneration and create a favorable immune milieu conducive to healing. Moreover, investigating the potential of exosomes in mitigating autoimmune responses or promoting tolerance in the context of tissue transplantation might open new avenues in regenerative medicine and beyond.

As we contemplate the future of exosome research, it is imperative to consider the long-term effects and potential risks. While short-term safety profiles have generally been favorable, comprehensive longitudinal studies are needed to assess the long-term impact of exosome treatments on multiple aspects such as tissue homeostasis, cellular senescence, and potential oncogenic risks. Therefore, employing advanced in vivo imaging techniques and molecular profiling methods to track the fate and effects of administered exosomes over extended periods is also essential.

Integrating exosome biology with emerging fields such as 3D bioprinting presents an exciting frontier. Incorporating exosomes into bioengineered scaffolds or using exosome-laden bioinks in 3D printing applications to enhance tissue regeneration and vascularization deserves further exploration. This approach could develop more complex, functional tissue constructs that better recapitulate native tissue architecture and function.

Another underexplored area is the exosome’s potential role in epigenetic reprogramming for tissue regeneration. Exosomes carry a variety of epigenetic modifiers, such as non-coding RNAs, which can alter the epigenetic landscape of recipient cells. Harnessing this epigenetic modulation capacity for regenerative purposes is a promising research direction. Profiling the epigenetic cargo of exosomes from different cell types, developing methods to selectively enrich exosomes with specific epigenetic modifiers, and investigating the long-term stability of exosome-induced epigenetic changes in regenerating tissues are crucial aspects to explore. Investigating the potential of exosomes in reprogramming somatic cells into induced pluripotent stem cells or directly into specific cell types needed for tissue repair, without genetic manipulation, could open new avenues for precise and efficient tissue regeneration.

To the best of our knowledge, the present study is the first review to discuss the applications of exosomes in regenerative medicine from the perspective of cellular sources, aiming to provide a reference for researchers to choose the cell type of exosome sources. The future research directions in exosome-based regenerative medicine are diverse and interconnected, necessitating a holistic approach that spans basic science, translational research, and clinical development. Addressing these multifaceted challenges could unlock the transformative potential of exosomes in regenerative medicine, ushering in a new era of personalized and cell-free therapies.

## Figures and Tables

**Table 1:** Examples of different exosomal dosages and exosomal administration routes.

Exosomal Species Origin	Cellular Source	Application/Effect	The Most Effective Dosage/Concentration	Administration Route	Refs.
Rats	BMSC	sciatic nerve crush injury	0.9 × 10^10^ particles/mL	in vitro administration	[35]
Human	ADSC	wound healing	200 μg/mL	local administration	[36]
Human		Alzheimer’s disease	4 × 10^8^ particles in saline (1 mL) two times per week	intranasally administration	[37]
Human	UCMSC	complex perianal fistulas	10 ug/100 uL per rat	local administration	[38]
Human	HUVEC	vascular repair	0.35–1.75 μg/mL	in vitro administration	[39]
		repair of intestinal structure and function in rats with severe burn injuries	200 μg/0.5 mL per rat	intraperitoneal injection	[40]

**Table 2:** The examples of exosome applications mentioned above.

Exosome Sources	Application Areas	Specific Effects		Refs.
BMSC	Bone and cartilage regeneration; wound healing; osteonecrosis of the Femeral Head; immunomodulation; myocardial infarction; nervous system injury	(+)	Osteogenesis; angiogenesis; keratinocyte and DF proliferation; astrocyte differentiation; BMSC proliferation and differentiation; NSC differentiation	[55,56,57,58,59,60,61]
		(−)	Inflammation; T cell proliferation; glial scar	
ADSC	Wound healing; scar prevention; osteoarthritis; immunomodulation; myocardial ischemia; hair loss; skin photoaging	(+)	Angiogenesis; adipogenesis; keratinocyte and DF proliferation; collagen synthesis; growth of hair follicles; dermal papilla cell proliferation	[55,57,70,71,72,73,74,75,76,77]
		(−)	Inflammation; fibrotic; cartilage degeneration; skin photoaging; T cell proliferation	
Human UCMSC	Wound healing; cutaneous regeneration; colitis; autoimmune uveoretinitis; GVHD	(+)	Keratinocyte and DF proliferation; intestinal lymphatic drainage improvement	[55,57,80,81,82,83,85]
		(−)	Inflammation; T cell proliferation; macrophage pyroptosis; lymphangiogenesis; endoplasmic reticulum stress in CD4^+^ T cells	
Human AMMSC	Liver fibrosis; nonalcoholic steatohepatitis; wound healing; hypoxia/ischemia-induced cerebral palsy; acute TSCI	(+)	Reepithelization; collagen synthesis; angiogenesis; axonal regeneration	[92,93,94,95,96,97]
		(−)	Inflammation; activation of Kupffer cell and hepatic stellate cell; excessive apoptosis; astrogliosis; blood-spinal cord barrier leakage; spinal cord edema	
Human CMMSC	Osteoarthritis	Could be uptaken by different types of cells isolated from tissues associated with osteoarthritis		[98]
Human CVMSC	Hypoxic placenta	(+)	Trophoblast migration and proliferation; placental vascular adaptation to low oxygen tension	[99,100]
Human DCMSC	Wound healing	(+)	Fibroblast proliferation, migration, and differentiation; fibroblast senescent state improvement; collagen deposition	[101]
		(−)	Oxidative stress	
DPSC	Wound healing; dental pulp-like tissue regeneration; molar defect; periodontitis; cerebral I/R injury; subarachnoid hemorrhage; sciatic nerve injury; mandibular bone defect	(+)	Odontogenic differentiation of DPSC; dentinogenesis; neuroprotection; SC proliferation; migration and secreting neurotrophic factors; osteogenesis	[106,107,108,109,110,111,112,113,114,115,116,117]
		(−)	Imbalance of Th17/Treg; inflammation; alveolar bone loss; neuronal apoptosis; microglial pyroptosis; brain edema	
M2	Fracture healing; wound healing; chronic rotator cuff tear; contusion SCI; calvarial bone defect	(+)	Macrophage polarization (M1 to M2); osteogenesis; angiogenesis; reepithelialization; collagen deposition; neurogenesis; tube formation, migration and proliferation of brain endothelial cell	[122,123,124,125,127,128,130]
		(−)	Inflammation; cellular senescence of BMSC	
Treg	Skin xenograft transplantation; kidney allotransplantation; orthotopic liver transplantation; oxygen-glucose deprivation/reperfusion; wound healing; acute myocardial infarction	(+)	Naive T cell converting into Treg; migration of human DF and HUVEC; macrophage polarization (M1 to M2)	[120,131,132,133,134,135,136]
		(−)	Inflammation; effector T cell proliferation; BV-2 microglia apoptosis; myocardial cell apoptosis	
NSC	Thromboembolic stroke; SCI; Alzheimer’s disease; wound healing	(+)	Neurogenesis; angiogenesis; neurite remodeling; autophagy activation of spinal cord neuron; human DF migration; tube formation of HUVEC	[137,138,139,140,141,142,143]
		(−)	Brain atrophy; neuronal apoptosis; lipopolysaccharide-induced nitric oxide production by macrophage; activation of microglia; inflammation; BBB leakage; aging	
Fibroblast	Wound healing; nerve defect; skin photoaging; atopic dermatitis	(+)	ECM formation; angiogenesis; collagen deposition and maturity; axon regeneration and functional recovery; Schwann cell-mediated peripheral neuron myelination;	[144,146,147,148,149,150,151,152]
		(−)	Scar formation; oxidative stress; inflammation; collagen degradation	
HUVEC	Wound healing; hypoxia/reoxygenation; flap transplantation; transient cerebral I/R; cranial defect; SCI	(+)	HUVEC and human cutaneous keratinocyte migration; angiogenesis; skin proliferation; reepithelialization; granulation tissue formation; vascularization; endothelial progenitor cell proliferation, tube formation, and invasion; nerve cell migration and invasion; osteogenesis; macrophage polarization (M1 to M2); osteogenic differentiation and migration of BMSC	[153,155,156,157,158,159,160,161,162]
		(−)	Inflammation; scar proliferation; endoplasmic reticulum stress; neural cell apoptosis	
SC	Nerve crush; nerve axotomy; OGD-injured motoneuron; optic nerve crush; denervated muscle atrophy: SCI; cranial defect; CMS-induced DRG injury;	(+)	Axonal regeneration; motoneuron repair; angiogenesis; injured neuron autophogy; mitophagy; BMSC osteogenesis; proliferation and multipotency of human dental pulp cell; proliferation of injured DRG cell	[147,163,164,165,166,167,168,169,170,171,172]
		(−)	Oxidative stress; inflammation; mitochondrial damage; senescence of injured DRG cell	

((+): promoting effects, (−): suppressing effects).

**Table 3:** Studies comparing different types of exosomes and their findings.

Species Origin	Exosomes	In Vitro Model and Findings		In Vivo Model and Findings		Refs.
		Ability/Effect	Comparing Findings	Ability/Effect	Comparing Findings	
Rats	SC-Exos vs. FC-Exos vs. NSC-Exos	The ability to induce BMSCs into SCs	SC-Exos ≥ NSC-Exos > FC-Exos > Control	NA	NA	[180]
Mice	BMSC-Exos vs. ADSC-Exos	Promotion of proliferation, migration, osteogenic differentiation, and chondrogenic differentiation ability of BMSCs	BMSC-Exos ≈ ADSC-Exos	The ability to accelerate bone-tendon injury healing in murine rotator cuff injury model	BMSC-Exos ≈ ADSC-Exos	[181]
Human	Small extracellular vesicles derived from stem cell from human exfoliated deciduous teeth (SHED-sEVs) vs. Small extracellular vesicles derived from DPSC (DPSC-sEVs)	Promotion of the proliferation, migration, and osteogenesis of periodontal ligament stem cells (PDLSCs)	SHED-sEVs > DPSC-sEVs	NA	NA	[182]
Human	BMSC-EVs vs. ADSC-EVs vs. UCMSC-EVs vs. Extracellular vesicles derived from dermal stem cell (DSC-EVs) vs. DPSC-EVs	Productivity	UCMSC-EVs > others	Biodistribution in full-sickness skin defect mouse models	Enrichment in the spleen, lungs, kidneys, and lymphonodus: UCMSC-EVs > others, in bone marrows: BMSC-EVs > others.	[104]
		Cell affinity	Immune cells and recipient cells in tissue regeneration: UCMSC-EVs > others, neuroblastoma cells: DPSC-EVs > others.	Wound-healing potential in full-sickness skin defect mouse models	BMSC-EVs ≈ CMSC-EVs ≈ DSC-EVs > ADSC-EVs ≈ DPSC-EVs	
		Drug loading/delivery capacity	UCMSC-EVs/DPSC-EVs > others			
Human	BMSC-Exos vs. ADSC-Exos vs. UCMSC-Exos	Suppressing glycolysis and pro-inflammatory cytokine release in LPS-treated macrophages	ADSC-Exos > others	Alleviating sepsis-induced ALI and systemic inflammation and improving survival of ALI mice	ADSC-Exos > others	[183]
Human	CDC-EVs vs. BMSC-EVs vs. ADSC-EVs	Upregulating the Arg1/Nos2 ratio of peritoneal Mϕ of thioglycolate-stimulated mice	CDC-EVs > MSC-EVs	Reduction in scar size and increase in infarct wall thickness in a mouse model of MI	CDC-EVs > MSC-EVs	[184]
Human	BMSC-Exos vs. ADSC-Exos vs. WJMSC-Exos	Enhancing neuronal differentiation, postponing neutrophil apoptosis, and PBMC proliferation.	BMSC-Exos > others	NA	NA	[57]
		Promoting angiogenesis	WJMSC-Exos > others			
Human	BMSC-EVs vs. ADSC-EVs	Promoting endothelial cell proliferation The effects on the most relevant cell types involved in skin wound healing	BMSC-EVs > ADSC-EVs	Accelerating wound closure in a mouse model of diabetic ulcers	ADSC-EVs > BMSC-EVs	[56]
		Promoting fibroblast, keratinocyte, and endothelial cell viability	BMSC-EVs > ADSC-EVs			
		Promoting migration of endothelial cells	ADSC-EVs > BMSC-EVs			
		Inducing vessel formation	ADSC-EVs > BMSC-EVs			
Human	ADSC-EVs vs. Extracellular vesicles derived from DF (DF-EVs)	Top 3 enriched biological pathways	ADSC-EVs: Positive regulation of macrophage cytokine production, type B pancreatic cell proliferation, and positive regulation of I-κB kinase/NF-κB signaling	NA	NA	[185]
			DF-EVs: non-canonical Wnt signaling pathway, Wnt signaling pathway, planar cell polarity pathway, and receptor-mediated endocytosis			
Human	BMSC-Exos vs. ADSC-Exos vs. UCMSC-Exos	Potential applications of exosomes in different fields via proteomics	BMSC-Exos: superior regeneration ability	NA	NA	[80]
			ADSC-Exos: immune regulation			
			UCMSC-Exos: tissue damage repair			
Human	BMSC-Exos vs. Exosomes derived from stem cells from the apical papilla (SCAP-Exos)	Different expression profiles of PIWI-interacting RNAs (piRNAs) and the related functions	The higher expression of piRNAs in BMSC-Exos: the regulation of apoptosis and osteogenic differentiation	NA	NA	[186]
			The higher expression of piRNAs in SCAP-Exos: metabolism, cell proliferation and differentiation, and other signaling pathways closely related to the development of teeth and the formation of bone tissue			
Human	BMSC-EVs vs. DPSC-EVs	Chemotactic capacity on endothelial cells	BMSC-EVs > ADSC-EVs, this difference was neutralized when the results were normalized for the higher particle secretion of BMSCs.	NA	NA	[187]
Human	BMSC-Exos vs. ADSC-Exos vs. UCMSC-Exos	Inducing primary DF proliferation	BMSC-Exos > others	NA	NA	[55]
		Stimulating keratinocyte migration	UCMSC-Exos > others			
Canine species	BMSC-EVs vs. ADSC-EVs	Productivity	BMSC-Exos > ADSC-Exos	NA	NA	[188]
		Differential function of exosomal proteins	BMSC-Exos: cell differentiation, cell organization and biogenesis, cellular component and movement, metabolic process, regulation of biological process, response to stimulus and transport			
Human	BMSC-Exos vs. ADSC-Exos	Suppressing the differentiation of CD4 + T cells into Th17 and the secretions of pro-inflammatory factors IL-17 and TNF-α	DPSC-Exos > BMSC-Exos	NA	NA	[108]
		Promoting the polarization of CD4 + T cells into Treg and increasing the release of anti-inflammatory factors IL-10 and TGF-β	DPSC-Exos > BMSC-Exos			
		Inducing apoptosis of CD4 + T cells	DPSC-Exos > BMSC-Exos			
Human	NSC-EVs vs. MSC-EVs	NA	NA	Improving cellular, tissue, and functional outcomes in middle-aged mouse TE stroke models	NSC-EVs > MSC-EVs	[137]
Human	BMSC-Exos vs. DPSC-Exos	Neuroprotective properties	BMSC-Exos ≈ DPSC-Exos	NA	NA	[109]

(NA: not applicable).
